# Aerobic Interval Training Partly Reverse Contractile Dysfunction and Impaired Ca^2+^ Handling in Atrial Myocytes from Rats with Post Infarction Heart Failure

**DOI:** 10.1371/journal.pone.0066288

**Published:** 2013-06-14

**Authors:** Anne Berit Johnsen, Morten Høydal, Ragnhild Røsbjørgen, Tomas Stølen, Ulrik Wisløff

**Affiliations:** K.G. Jebsen Center of Exercise in Medicine, Department of Circulation and Medical Imaging, Faculty of Medicine, Norwegian University of Science and Technology, Trondheim, Norway; Cardiovascular Research Institute Maastricht, Maastricht University, The Netherlands

## Abstract

**Background:**

There is limited knowledge about atrial myocyte Ca^2+^ handling in the failing hearts. The aim of this study was to examine atrial myocyte contractile function and Ca^2+^ handling in rats with post-infarction heart failure (HF) and to examine whether aerobic interval training could reverse a potential dysfunction.

**Methods and results:**

Post-infarction HF was induced in Sprague Dawley rats by ligation of the left descending coronary artery. Atrial myocyte shortening was depressed (p<0.01) and time to relaxation was prolonged (p<0.01) in sedentary HF-rats compared to healthy controls. This was associated with decreased Ca^2+^ amplitude, decreased SR Ca^2+^ content, and slower Ca^2+^ transient decay. Atrial myocytes from HF-rats had reduced sarcoplasmic reticulum Ca^2+^ ATPase activity, increased Na^+^/Ca^2+^-exchanger activity and increased diastolic Ca^2+^ leak through ryanodine receptors. High intensity aerobic interval training in HF-rats restored atrial myocyte contractile function and reversed changes in atrial Ca^2+^ handling in HF.

**Conclusion:**

Post infarction HF in rats causes profound impairment in atrial myocyte contractile function and Ca^2+^ handling. The observed dysfunction in atrial myocytes was partly reversed after aerobic interval training.

## Introduction

Impaired cardiomyocyte contractility and Ca^2+^ handling are hallmarks of left ventricular contractile dysfunction. Reduced Ca^2+^ transients, slowed transient decay, increased diastolic Ca^2+^, reduced sarcoplasmic reticulum (SR) Ca^2+^ stores, reduced function of the SR Ca^2+^ ATPase (SERCA) 2a, increased Na^+^/Ca^2+^ exchanger (NCX) activity and increased diastolic SR Ca^2+^ leak are commonly observed in cardiomyocytes from failing hearts [1]. Despite that atrial myocytes contribution to ventricular filling obviously affect the subsequent ejection of blood from the heart, there are limited data on contractile function and Ca^2+^ handling in atrial myocytes from failing hearts. Disruption of the rhythmic beating of atrial myocytes can lead to life-threatening conditions such as atrial fibrillation (AF), the most common cardiac arrhythmia in clinical practice [2], [3]. In a recent study involving dogs with congestive heart failure (HF) decreased atrial cell shortening, abnormal SR Ca^2+^ handling and changes in Ca^2+^ regulatory proteins was observed [4]. Impaired Ca^2+^ handling and atrial myocytes contractile dysfunction was also observed in goats with dilated atrias [5].

Exercise training has been reported to improve left ventricular function after post-infarction HF in patients [6]. In addition, exercise is found to improve cardiomyocyte function and Ca^2+^ handling in rats with post-infarction HF [7], [8]. To our knowledge it is not known whether there are any beneficial effects of exercise on atrial myocyte function and Ca^2+^ handling in HF. To address this issue we compared contractile function and Ca^2+^ handling in atrial myocytes of sham-operated rats and rats with post-infarction HF, and studied the effects of aerobic interval training.

## Methods

### Rat Model of Heart Failure and Exercise Training

The Norwegian council for Animal Research approved the study, which was in accordance with Guide for the Care and Use of Laboratory Animals (National Institutes of Health Publication No. 85-23, revised 1996).

Female Sprague Dawley rats were randomized to either sham operation or myocardial infarction (MI) surgery. MI was induced by ligation of the left coronary artery, as previously described [9]. Briefly, rats were anesthetized with 5% isoflurane in a closed chamber, intubated and ventilated with 1.5% isoflurane in a 70% O_2_ 30% N_2_O mixture. After left thoracotomy and pericardium opening, the descending artery was ligated with a polyester suture (Ethibond 6-0, needle Rb-2, Ethicon; Norderstedt, Germany). Sham operated rats were subjected to the same surgical procedures except the ligation. Buprenorfin (0.04 mg/kg) was injected subcutaneous during the surgery and repeated 8 hours after surgery for relieving the pain. After 1 and 4 weeks, MI operated rats were examined by echocardiography to determine the extents of MI. Previous echocardiography examinations of MI rats in our laboratory have shown that infarct size is a strong predictor for remodeling and HF where infarct size >40% confirmed HF progression [7], [9]. Rats with small MIs (<40%) were excluded from further study. Rats with large MIs (HF-rats, MI >40%) were randomized to aerobic interval training (n = 9) or to a sedentary group (n = 9) and both groups were compared to sham sedentary group (n = 9). To determine maximal oxygen uptake (*V*O_2max_), rats ran on a customized treadmill in a metabolic chamber, as previously described [10]. Aerobic interval training was performed as uphill running (25°), alternating between 4 min at 85%–90% of *V*O_2max_ and 2 min at 50% of *V*O_2max_ for 60 min/day, 5 days/week, for 8 weeks. VO_2max_ was determined every second week to account for the improved training status, then the workload was adjusted to keep high intensity intervals at 85%–90% of VO_2max_.

After the intervention period, the rats were sacrificed during anesthesia; breathing 5% isoflurane in a closed chamber, thereafter breathing 1.5% isoflurane in a 70% O_2_ 30% N_2_O mixture while the heart was removed and left atria was selected for cell experiments. The estimated infarct size from echocardiography was confirmed in the excised heart as previously described [9]. During the intervention period, one HF sedentary and one HF interval trained rat died (not exercise related).

### Atrial Myocyte Isolation and Ca^2+^ Measurements

Left atrial cells from rat were isolated using a modified mouse model protocol [11]. After removal, the hearts were kept in ice-cold cell isolation buffer (130 mM NaCl, 5.4 mM KCL, 0.5 mM MgCl_2_, 0.33 mM NaH_2_PO_4_, 22 mM glucose, 50 µU/ml bovine insulin (I-5500, Sigma), 25 mM HEPESNaOH (pH = 7.4)) with 0.4 mM EGTA and immediately canulated through aorta and retrogradely perfused (7.5 ml/min, 37°C) with isolation buffer containing 0.4 mM EGTA for 2–4 min. Then the hearts were perfused with the enzyme solution containing isolation buffer supplemented with 0.048 mM CaCl_2_ and 1 mg/ml collagenase (Type II, Worthington, 295 U/mg). From the digested hearts (10–15 minutes perfusion) left atria were removed, cut into 3–5 pieces, and further digested by gentle stirring for 5–10 min in fresh enzyme solution until myocytes appeared. Tissue chunks were then transferred to isolation buffer containing 0.096 mM CaCl_2_ and 10 mg/ml 0.1% bovine serum albumin, cut into as small pieces as possible and mechanically agitated with a pipette. The cell suspension was centrifuged at 40×g for 2 minutes in a 15 ml plastic centrifuge tube, the supernatant was gently removed and the cell pellet was resuspended in 2 ml of isolation buffer with 0.026 mM CaCl_2_.

For Ca^2+^ measurements cells were resuspended in HEPES solution (144 mM NaCl, 5.4 mM KCl, 0.3 mM NaH_2_PO_4_×H_2_O, 1.0 mM MgCl_2_×6 H_2_O, 5.0 mM Hepes, 11.1 mM glucose) containing 1.8 mM CaCl_2_. Fura-2/AM-loaded (20 minutes in 2 µM, Molecular Probes, Eugene, OR) cardiomyocytes were field stimulated by bipolar electrical pulses at 2 Hz on an inverted epifluorescence microscope (Nikon TE-2000E, Tokyo) with a 40×1.3NA oil-immersion objective. 8–10 cardiomyocytes were analyzed for each measurement. Cell shortening was measured by video-based sarcomere spacing (Ionoptix, Milton) and intracellular Ca^2+^ concentration was measured by counting 510 nm emission with a photomultiplier tube (PMTACQ, IonOptix, Milton, MA) after exciting with alternating 340 and 380 nm wavelengths (*F^340/380^* ratio) (Optoscan, Cairn Research, Kent, UK). Total SR Ca^2+^ content was measured by assessing peak Ca^2+^ amplitude after rapidly applying Caffeine (10 mM) to the perfusion solution by a pipette placed directly above the cardiomyocyte. The application of caffeine was performed immediately after stopping the electrical stimulation in normal HEPES solution. Diastolic Ca^2+^ cycling was quantified with the use of rate constants of Ca^2+^ removal during twitch-induced stimulation, sustained caffeine stimulation in physiological HEPES solution and sustained caffeine stimulation in a solution containing 0Na^+^/0Ca^2+^. During a regular twitch-induced Ca^2+^-transient in normal physiological HEPES solution, Ca^2+^ is removed by the SERCA2a, NCX, and the plasma membrane Ca^2+^ ATPase (PMCA), and the rate constant of Ca^2+^ decline in this situation (K_tw_) can be described as the sum of the rate constants associated with each efflux mechanism. During caffeine-induced Ca^2+^-transients, the contribution from SERCA2a is abolished, and the decay rate constant thus depends only upon NCX and PMCA. To derive the rate constant of NCX (K_NCX_), the rate constant of Ca^2+^ removal during caffeine-induced Ca^2+^ transients in a solution containing 0Na^+^/0Ca^2+^ was recorded and subtracted from the rate constant in the presence of these ions (Caffeine+regular HEPES) [12]. To quantify the contribution from SERCA2a, a simple model was used based on the following assumptions: SERCA2a transport rate is K_SERCA2a_ = K_tw_ – K_NCX_, and the relative contribution by SERCA2a is K_SERCA2a_/K_TW._

Diastolic SR Ca^2+^ leak was assessed using a modified protocol after Shannon et al [13]. After recording contractile function and Ca^2+^ handling during twitch-induced transients in HEPES 1.8 mM Ca^2+^ solution, the electrical field stimulation was ceased and quiescent cardiomyocytes were perfused with a physiological solution containing 0 Na^+^/0Ca^2+^. The 0Na^+^/0Ca^2+^ solution prevents the Na^+^ - Ca^2+^ exchange, which is the primary Ca^2+^-influx and efflux mechanism at rest. After recording the Ca^2+^ level in the quiescent cardiomyocyte for 40 seconds we rapidly switched to Caffeine +0Na^+^/0Ca^2+^. When Ca^2+^ levels were close to baseline, caffeine perfusion was replaced by HEPES perfusion and cardiomyocytes were stimulated at 1 Hz until stable Ca^2+^ transients were restored (approximately 20 seconds). Subsequently, perfusion was switched to a 0Na^+^/0Ca^2+^+Tetracaine (1 mM) solution whereupon diastolic Ca^2+^ level were measured for another 40 seconds, again followed by a 0Na^+^/0Ca^2+^+caffeine-induced transient. The quantitative difference in diastolic Ca^2+^ level between the 0Na^+^/0Ca^2+^ solution with and without tetracaine represents the absolute SR Ca^2+^ leak, since 0Na+/0Ca^2+^ prevents the NCX Ca^2+^ influx and efflux across the sarcolemma and tetracaine inhibits Ca^2+^ movement across RyR2. Diastolic SR Ca^2+^ leak was quantified in relation to total SR Ca^2+^ content.

To determine the influence of Ca^2+^/calmodulin-dependent protein kinase II (CaMKII) and protein kinase A (PKA) on diastolic SR Ca^2+^ leak from the RyR2, the CaMKII inhibitor (Autocamtide 2-related inhibitory peptide, AIP, 1 µM, Sigma Aldrich, MO) and PKA inhibitor (H-89, 3 µM, Sigma Aldrich, MO) were used. Pre-incubation of cardiomyocytes with AIP/H-89 started 1 hour prior to experiments. During the experimental protocol, the inhibitors were added to the perfusion solution to avoid potential washout of inhibitors.

### Statistics

Data are presented as mean ± SD. One-way ANOVA with Bonferroni post-hoc test adjusted for multiple comparisons was used to identify statistical differences between the groups. P<0.05 was considered statistical significant.

## Results

### VO_2max_

**Figure 1:**
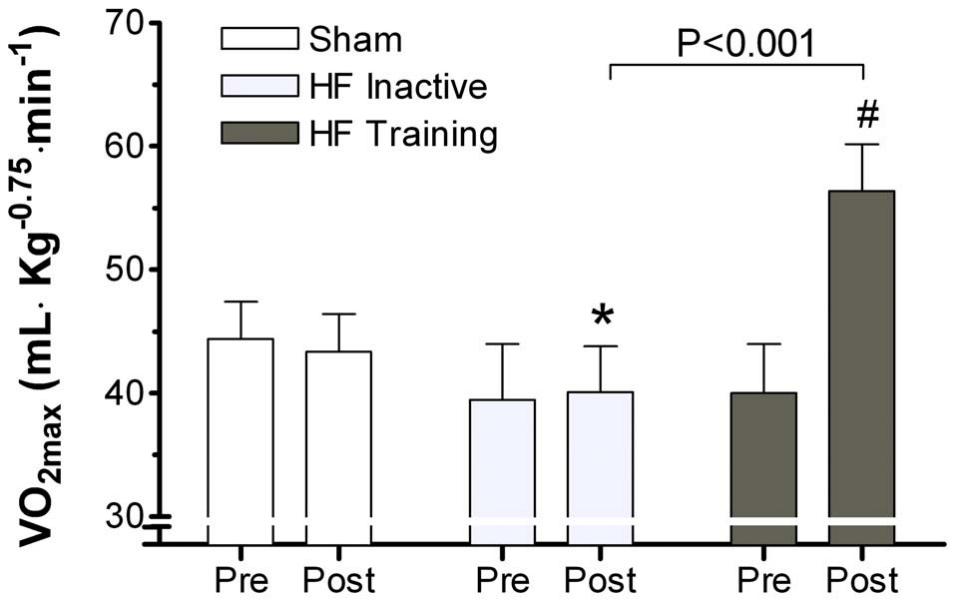
Averaged data of pre- and post –measurements of VO_2max_. **Pre- and post-values in sham and HF-inactive groups refers to basal measurements and time-dependent changes after 8 weeks (the period with training in exercise group).** VO_2max_ was lower in HF-rats compared to sham, referring to the post-values (p<0.05). Aerobic interval training in HF-rats improved VO_2max_ to a level above inactive sham rats (p<0.001). * *p<0.05* versus sham sedentary (post values), *# p<0.001* versus HF training (pre values).

VO_2max_ was 7.6% lower in rats with HF compared to sham (p<0.05). Aerobic interval training improved VO_2max_ by 41.0%, which was higher than that observed in inactive sham-rats (p<0.001, Figure 1).

### Atrial Myocyte Contractile Function

**Figure 2:**
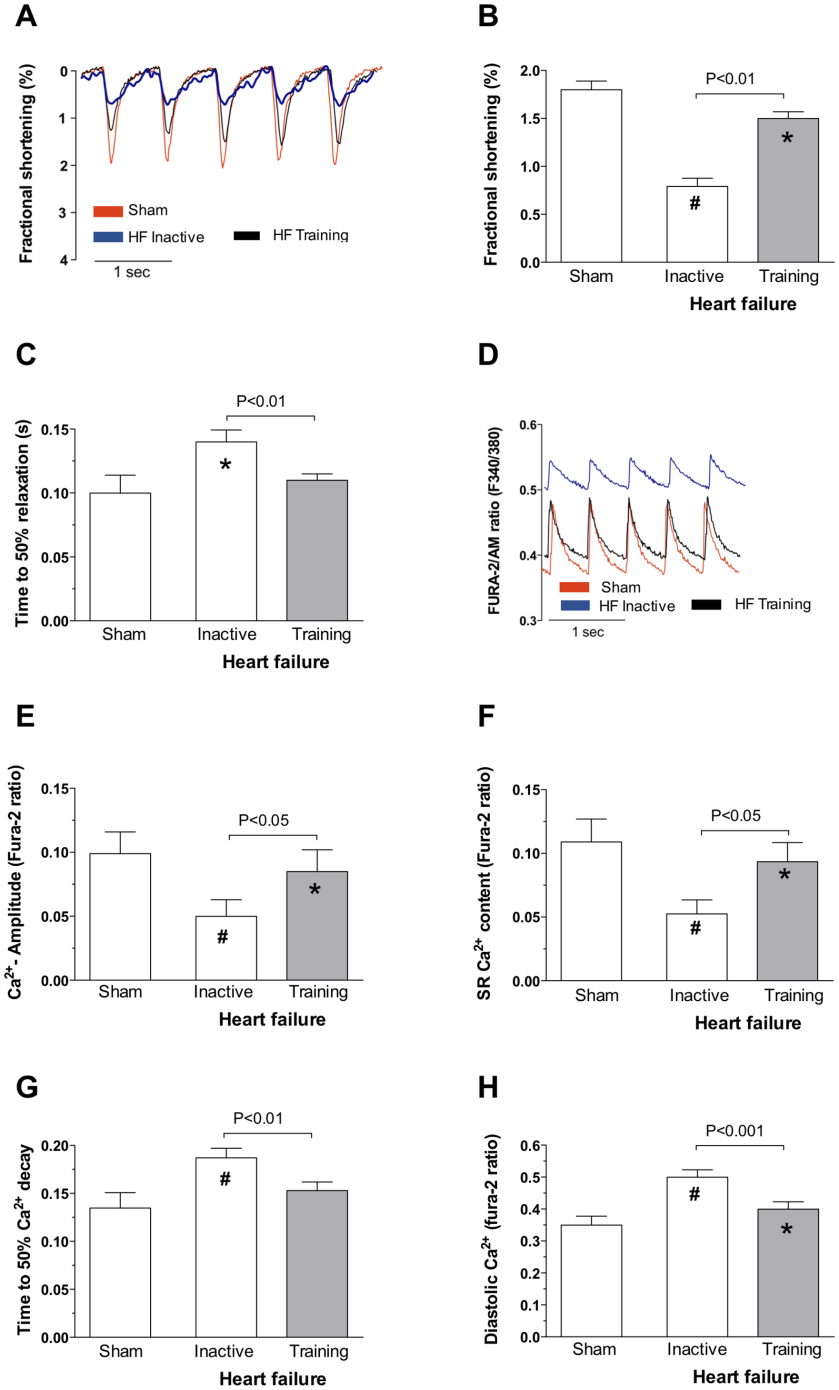
Representative sample traces of cardiomyocyte fractional shortening (A) and Fura-2 ratio (D) from sham, HF-inactive and HF-trained rats. Averaged data of myocyte fractional shortening (B), time to 50% diastolic relaxation (C), Ca^2+^ amplitude (E), SR Ca^2+^ content (F), time to 50% Ca^2+^ decay (G) and diastolic Ca^2+^ (H). All averaged data presented as mean values ± SD. * *p<0.05* versus sham sedentary, *# p<0.001* versus sham sedentary.

Atrial myocyte fractional shortening was 56.1% lower in rats with post-infarction HF compared with that observed in sham (p<0.001, Figure 2B). Aerobic interval training improved atrial myocyte shortening by 89% in HF-rats compared with HF-rats that remained inactive (p<0.01). Still, atrial myocyte shortening in interval trained HF-rats was lower (p<0.05) than that observed in inactive sham-rats (Figure 2B). Diastolic function, measured as time to 50% re-lengthening, was prolonged in atrial myocytes from HF-rats (140±9.3 ms, p<0.05) whereas aerobic interval training reduced time to 50% re-lengthening to levels of sham-rats (110±5.0 ms, Figure 2C).

### Ca^2+^ Handling

In line with contractile function, atrial myocyte Ca^2+^ handling was depressed after HF and improved after interval training. In inactive HF-rats the Ca^2+^ transient amplitude was 49.5% lower compared with that observed in sham-rats (p<0.001, Figure 2C). Aerobic interval training increased the Ca^2+^ amplitude by 70% in HF-rats (p<0.05), but it remained lower (p<0.05) than that observed in sham (Figure 2E). Similarly, SR Ca^2+^-load was 48% lower (p<0.001) in inactive HF-rats vs. sham, and increased by 78% (p<0.05) after aerobic interval training, but still remained 15% lower (p<0.05) than that observed in sham (Figure 2F). In line with a slower atrial myocyte re-lengthening, time to 50% Ca^2+^ decay was slower in inactive HF-rats compared to sham-rats (187±1.0 ms vs. 135±1.6 ms, p<0.001, Figure 2G). In HF-rats, aerobic interval training improved Ca^2+^ removal to levels above inactive sham-rats (153±9.0 ms vs 135±1.6 ms, p<0.05).

The intracellular diastolic Ca^2+^ level was higher in atrial myocytes from inactive HF-rats compared to sham (p<0.001, Figure 2H). Aerobic interval training lowered the diastolic Ca^2+^ level but it still remained higher than that observed in sham-rats (p<0.05).

**Figure 3:**
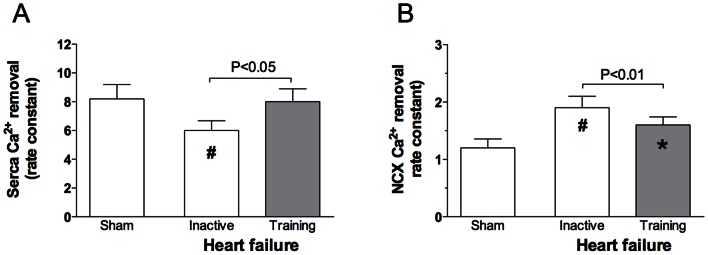
Averaged data of rates of decay of the twitch- and Caffeine induced Ca^2+^ transient indicating the SERCA2a function (A) and NCX function (B). All data presented as mean values ± SD. * *p<0.05* versus sham sedentary, *# p<0.001* versus sham sedentary.

The activity of SERCA2a was 26.8% lower in atrial myocytes from HF-rats compared to sham (p<0.001, Figure 3A). Aerobic interval training improved SERCA2a-function in HF-rats to a level comparable to that observed in atrial myocytes from sham. The activity of the NCX was increased by 58.3% in HF-rats compared to sham (p<0.001, Figure 3B). Aerobic interval training decreased NCX activity in HF-rats to a level intermediate between sham (p<0.05) and inactive HF-rats (p<0.01).

### Diastolic SR- Ca^2+^ Leak

**Figure 4:**
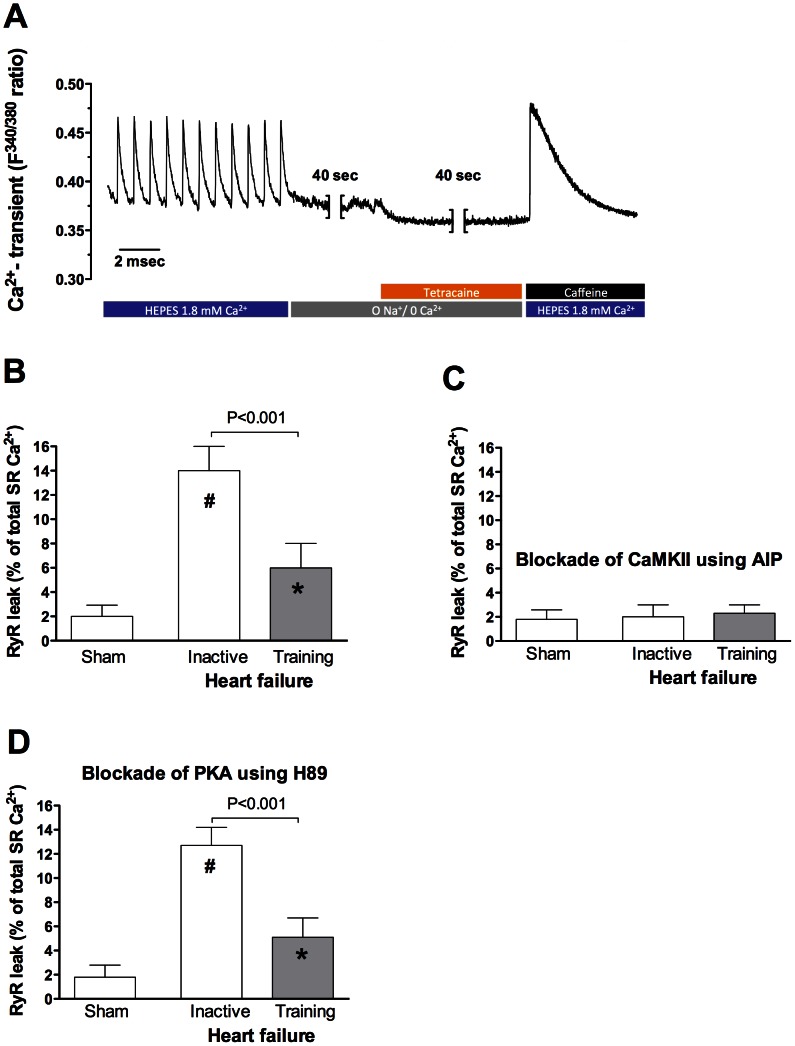
Illustration of the SR Ca^2+^ leak protocol (A), mean data of SR Ca^2+^ leak normalized to SR Ca^2+^ content (B), and mean data of SR Ca^2+^ leak after CaMKII inhibition (AIP) (C) and PKA inhibition (H89) (D). * *p<0.05* versus sham sedentary, *# p<0.001* versus sham sedentary.

We found a 7-fold increase in diastolic SR Ca^2+^ leak in atrial myocytes from HF-rats compared to that observed in sham rats (p<0.001, Figure 4A–B). Aerobic interval training in HF-rats reduced diastolic SR Ca^2+^ leak by 57.1% (p<0.001), but the Ca^2+^ leak was still significantly higher compared to sham rats (p<0.05). Pharmacological inhibition of CaMKII by AIP reduced Ca^2+^ leak in atrial myocytes from inactive and exercise trained HF-rats to that observed in sham (Figure 4C), whereas PKA inhibition by H-89 had no effect on diastolic SR Ca^2+^ leak (Figure 4D).

## Discussion

This is the first study to report contractile dysfunction and impaired Ca^2+^ handling in atrial myocytes from rats with post-infarction HF and to demonstrate the therapeutic potential of aerobic interval training in reversing the atrial myocyte dysfunction.

### Impaired Atrial Cardiomyocyte Ca^2+^ Handing in HF

Reduced fractional shortening and prolonged time to diastolic re-lengthening in HF-rats were paralleled by reduced Ca^2+^ amplitude and slower Ca^2+^ decay. The reduced Ca^2+^ amplitude in this model was associated with reduced SR Ca^2+^ content. SR Ca^2+^ content is determined by diastolic SR Ca^2+^ uptake by SERCA2a, trans-sarcolemmal Ca^2+^ fluxes (mainly by the NCX) and diastolic Ca^2+^ leak over the RyR2 [14]. In the present study, the reduced SERCA2a function, increased NCX function and increased diastolic SR Ca^2+^ leak over the RyRs explain the reduced SR Ca^2+^ content. The observed impairment in atrial Ca^2+^ handling resembles previously reported HF-induced changes in ventricular cardiomyocytes [1], [15]–[17], that are likely to contribute to contractile dysfunction and increased propensity to promote arrhythmias [18], [19].

In the current study, the CaMKII inhibitor AIP decreased the SR Ca^2+^ leak in HF-rats while H89 that inhibits PKA had no effect. This suggests that Ca^2+^ release channel dysfunction is caused by CaMKII-dependent RyR2 hyperphosphorylation. This is consistent with data from several HF models, in which CaMKII-dependent increase in RyR2 phosphorylation was associated with higher ventricular myocyte SR Ca^2+^ leak [19]–[22]_ENREF_19 and also with data showing no PKA-dependent SR Ca^2+^ leak [20]. In agreement with our findings, decreased SR Ca^2+^ content was shown in two goat models of atrial dysfunction [5]. The reduced SR Ca^2+^ content was associated with a reduced reuptake of Ca^2+^ into SR by SERCA2a in addition to increased SR Ca^2+^ leak during diastole. Furthermore, despite not directly assessed in our study, the data from goat showing CaMKII mediated RyR2 hyperphosphorylation as mediator of SR Ca^2+^ leak [5], is in agreement with our data showing a reduced SR Ca^2+^ leak by inhibiting CaMKII with AIP. On the other hand, in contrast to the Ca^2+^ handling abnormalities we identified, Yeh et al [4] reported increased SR Ca^2+^ load, associated with increased Ca^2+^ entry (*I*_CaL_) and increased CaMKII phosphorylation of phospholamban (PLB) in a rapid ventricular tachypacing induced dog HF model. In its dephosphorylated state PLB inhibits SERCA2a. Phosphorylation by CaMKII (or PKA) dissociates PLB from SERCA2a increasing Ca^2+^ uptake into SR [23]. Data from the HF dog model are, however, not directly comparable to ours since HF induced by ventricular tachypacing differs from post myocardial infarction HF. It is not easy to pinpoint the exact differences, but it is well known that the balance of Ca^2+^ fluxes are significant different between dogs and rats (e.g. the integrated Ca^2+^ fluxes during twitch relaxation of the cardiomyocyte in rats depends on 92% SERCA2a and 7% NCX, whereas in dogs this relationship is 70%/28%, respectively) [24]. In addition, the data of increased intracellular Ca^2+^ loading from that model is not unexpected, since rapid pacing of the heart normally would cause this effect of increased Ca^2+^ loading of the cardiomyocyte [24], [25]. We observed functional reduction in the rate of Ca^2+^ removal by SERCA2a in the present study, though we did not measure the phosphorylation level of molecular determinants. Being as how earlier studies on goats have shown that PLB phosphorylation was reduced [5] we speculate that reduced phosphorylation of PLB could have contributed to suppression of SERCA2a Ca^2+^ uptake in the present study.

Alterations in Ca^2+^ homeostasis in HF may be a predisposing factor to atrial arrhythmic activity [26]. Hyperphosphorylation of RYR2 and the consequent Ca^2+^ leak from SR has been shown to be an important contributor to arrhythmogenesis [27], [28]. Reduction in SR Ca^2+^ uptake as well as SR Ca^2+^ leak through RyR2 contributes to accumulation of diastolic Ca^2+^ [29]. Increased diastolic Ca^2+^ concentration enhances the NCX activity and inward Na^+^ current during Ca^2+^ extrusion promoting delayed afterdepolarizations (DADs) and triggered activity [30], which can initiate or maintain AF. Furthermore, diastolic Ca^2+^ accumulation may induce diastolic dysfunction by activation of contractile proteins, preventing full relaxation of myofilaments [31]. In human AF it has been observed larger spontaneous Ca^2+^ release from SR [32], [33] together with enhanced NCX activity [33], which is consistent with data from our HF-rat model. In addition expression levels of NCX are reported to be upregulated in patients with AF [33]–[35]. Several studies have reported increased activity of NCX linked to increased expression of the protein in ventricular myocytes from human failing hearts [36], [37] and in experimental HF models [38]. It is important to note that AF was not characterized in our HF-rat model, but it seems reasonable to suggest that the Ca^2+^ handling abnormalities in this model may be of main importance for development of AF.

### Therapeutic Potential of Aerobic Interval Training in Restoring Atrial Cardiomyocyte Function

We demonstrate here for the first time that aerobic interval training improved atrial myocyte contractile function in HF-rats. It has previously been shown that such training programs improve contractile function in healthy animals [39], [40], as well as improving ventricular cardiomyocyte contractility in postmyocardial infarction HF [7]. Despite pathological remodelling and reduced contractile function, atrial myocytes maintain the ability to respond to exercise training by partly reversing the detrimental effects of HF.

We suggest that the improved atrial myocyte Ca^2+^ handling after exercise in HF-rats explains the observed increase in myocyte contractility. The normalized SERCA2a activity, reduced NCX activity, and reduced SR Ca^2+^ leak explains the increased Ca^2+^ amplitude and SR Ca^2+^ content, thereby contributing to the observed improvement in contractile function. Similarly, the normalized ventricular contractility in trained HF-rats was explained by increased rates of rise and decay of the Ca^2+^ transient associated with normalized NCX and SERCA2a [7]. Previous data from our group has also shown that exercise training reversed contractile abnormalities in diabetic mice, due to the aforementioned mentioned mechanisms [41]. In addition to the reversed contractile dysfunction in HF-rats we suggest that the shift in Ca^2+^ removal from the outer plasma membrane to the SR and the consequent decrease in Na^+^ current through the NCX constrain the ability to produce DADs, and likely decrease the susceptibility to triggered activity and arrhythmias.

As discussed above, we suggest that increased CaMKII activity in HF explains the increased diastolic Ca^2+^ leak in the present study. This explanation is in line with observation of increased phosphorylation level of CaMKII in diabetic cardiomyocytes showing abnormally high Ca^2+^ leak [41]. Exercise training was found to reverse the hyperphosphorylation of CaMKII in this diabetic model and is consistent with our data showing that training abolished the high SR Ca^2+^ leak. The reduced Ca^2+^ leak in the present study is suggested to be explained by training-induced dephosphorylation of CaMKII. In several HF models, inhibition of CaMKII has been shown to abolish SR Ca^2+^ leak [20], [42], as was observed in our model.

### Conclusion

This study increases our understanding of how atrial myocyte contractile dysfunction in post-infarction HF is associated with major impairment in Ca^2+^ handling, whereas aerobic interval training can restore the dysfunction via improved Ca^2+^ handling. In future studies this model can help us to elucidate important mechanisms behind HF as a substrate that promotes AF, and also the effect of training as a treatment.
